# Does dissatisfaction with, or accurate perception of overweight status help people reduce weight? Longitudinal study of Australian adults

**DOI:** 10.1186/s12889-019-6938-3

**Published:** 2019-05-22

**Authors:** Xiaoqi Feng, Andrew Wilson

**Affiliations:** 1 Population Wellbeing and Environment Research Lab (PowerLab), School of Health and Society, Faculty of Social Sciences, University of Wollongong, Wollongong, NSW 2522 Australia; 2 Menzies Centre for Health Policy, School of Public Health, the Faculty of Medicine and Health, The University of Sydney, Sydney, Australia; 3 The Australian Prevention Partnership Centre, The Sax Institute, Sydney, Australia

**Keywords:** Body mass index, Weight misperception, Disadvantage, Longitudinal

## Abstract

**Background:**

With studies around the world suggesting a large proportion of people do not recognise that they are overweight (or feel satisfied with being overweight), this fuels the view that such ‘misperceptions’ need to be ‘corrected’. However, few longitudinal studies have examined the consequences of under-perceived weight status, nor over-perceived weight status (when a person feels overweight when they are not) and weight-related satisfaction on trajectories in body mass index (BMI).

**Methods:**

Five-year BMI trajectories were examined among 8174 participants in an Australian nationally representative cohort. Each person was classified into groups according to their neighbourhood socioeconomic circumstances, baseline BMI and answers to *“how satisfied are you with your current weight?”* and “*do you consider yourself to be… acceptable weight / underweight / overweight?”* Gender-specific multilevel linear regressions were used to examine five-year BMI trajectories for people in each group, adjusting for potential confounders.

**Results:**

At baseline, weight-related dissatisfaction and perceived overweight were generally associated with higher mean BMI for men and women, regardless of whether they were classified as ‘normal’ or overweight by World Health Organization (WHO) criteria. Mean BMI did not decrease among people classified as overweight who perceived themselves as overweight, or expressed weight-related dissatisfaction, regardless of where they lived. Among men and women with ‘normal’ BMI at baseline but expressing weight-related dissatisfaction, mean BMI increased disproportionately among those living in disadvantaged areas compared to their counterparts in affluent areas. Similarly, mean BMI rose disproportionately among people in disadvantaged areas who felt they were overweight despite having a ‘normal’ BMI by WHO criteria, compared to people with the same over-perceptions living in affluent areas. These differences exacerbated pre-existing socioeconomic inequities in mean BMI.

**Conclusions:**

No evidence was found to suggest accurate recognition of overweight or expressing weight-related dissatisfaction leads to a lower BMI. However, there was evidence of an increase in mean BMI among people who felt dissatisfied with, or over-perceived their ‘normal’ weight, especially in socioeconomically disadvantaged areas. Correction of under-perceptions may not drive weight loss, but circumstances contributing to over-perception and dissatisfaction with weight status may contribute to increased weight gain and exacerbate socioeconomic inequities in BMI.

**Electronic supplementary material:**

The online version of this article (10.1186/s12889-019-6938-3) contains supplementary material, which is available to authorized users.

## Background

Weight gain among people living in disadvantaged neighbourhoods in high-income countries is comparatively higher and begins earlier than their peers in more affluent areas, especially for women [1–3]. Some longitudinal studies of adults in Australia [4] and the US [5] report comparatively greater weight gain over time among people who considered their weight status as ‘normal’, but were actually overweight or obese by World Health Organization (WHO) criteria. Evidence suggests under-perception of weight status is common [6–13]. Conventional wisdom suggests that correction of this ‘under-perception’ is a pre-requisite for behavioural change to achieve weight loss, or the slowing of the rate of weight gained over time [14, 15]. Evidence indicates there has been increased efforts by health professionals to correct under-perception of weight status in the US over the last 15 years [16].

However, some evidence suggests that under-perception of overweight status may be sometimes favourable. A recent systematic review found evidence to indicate that people who perceive their weight status as overweight were likely to gain more weight over time, despite also being more likely to attempt weight loss behaviours [17]. Studies of children have reported under-perception of overweight status to be associated with lower future weight gain [18] and lower blood pressure trajectories [19]. Meanwhile, there are some other studies that report problems with over-perception, or when people of ‘normal’ weight by WHO criteria perceive themselves as overweight or obese. Over-perception is common in some groups [20] and has been linked to future weight gain in adults [21, 22] and also contributing factors, such as increased psychological distress [23]. Similar findings have been reported in children and adolescents [24, 25]. Rather than being detrimental, under-perception of weight status may sometimes be protective (or indicative of the presence of some other protective factor, such as positive affect) against weight-related stigma and media-driven portrayals of idealised body size, which have been suggested to lead to heightened stress and maladaptive behaviours that contribute to weight gain [26, 27].

Satisfaction with current weight status has also been suggested to play a role in determining future weight loss. Studies have shown that body dissatisfaction and concern over weight and body shape intensify across adolescence [28], but then remain largely stable (particularly for women) for the rest of the lifecourse, despite physical changes associated with ageing [29]. Much of the research on body satisfaction and weight gain has been conducted on adolescents, with many studies finding more weight-related dissatisfaction among overweight adolescents to be predictive of future weight gain and maladaptive behaviours such as binge eating [30–34]. These findings run counter to belief that dissatisfaction with overweight status is a necessary precursor for positive change.

The abovementioned evidence provides interesting but conflicting perspectives on whether correction of under-perceived weight status ought to be mainstream policy. On one hand, correction of under-perception may be seen to enable people to take action and perhaps, in some contexts, receive additional support services (e.g. enrolment in a behavioural change program). But on the other, the psychosocial stress associated with being labelled as overweight or obese, even if a person is overweight by WHO criteria, may have unintended consequences, such as compounding body dissatisfaction. Since the majority of evidence is from the US, more studies are warranted to understand if either of these duelling hypotheses are supported in other contexts. It is possible that evidence for both hypotheses within the same national context may be available due to heterogeneity of experience between population sub-groups, such as well-known differences in weight-related stigma and discrimination experienced between men and women [35, 36].

Furthermore, it is plausible that different experiences will manifest across strata of area-level socioeconomic circumstances due to variation in the visual normalisation of overweight status [37]. In communities where being overweight or obese by WHO criteria is the norm, visual normalisation theory suggests that the BMI threshold by which people judge themselves to be overweight is raised [37]. In many countries the prevalence of overweight and obesity is at or above 60%, and within those countries overweight and obesity tend to be higher in socioeconomically disadvantaged areas [38]. This suggests the possibility of visual normalisation patterned geographically within countries. Recent studies report under-perception is more common among adults in socioeconomically disadvantaged areas [39] and in children of families with low educational attainment [40], perhaps because of visual normalisation [37] of larger body sizes. This may exacerbate the effect of other factors that contribute to the ‘obesogenic’ environment in disadvantaged areas, such as higher ratios of unhealthy to healthy food outlets [41] and visual cues that stimulate appetite for unhealthy food [42].

Accordingly, the purpose of this study was to examine five-year trajectories in body mass index (BMI) among male and female Australian adults stratified by their WHO criteria-defined weight status at baseline. Differences in BMI trajectories in each of these groups were examined in relation to each person’s perception of and satisfaction with their baseline weight status, as well as the potential for effect modification by area-level socioeconomic circumstances.

## Methods

### Data

Data analysed in this study was extracted from the “Household, Income and Labour Dynamics in Australia” (HILDA). Details of HILDA are already published [43]. In brief, HILDA is a nationally representative sample of approximately 15,000 individuals in 7000 households collected annually. A longitudinal sample of 4386 men and 3788 women aged > 15 years with complete BMI data in 2007 and 2012 was selected. These waves were selected as 2007 was the first year in which questions of perceived weight and weight-related satisfaction were asked, while data from 2012 was the most recent wave that was available at the time of analysis. Participants considered ‘underweight’ by WHO criteria (BMI < 18.5 kg/m^2^) were omitted as our focus was on contrasting people who were overweight or obese (BMI > 25 kg/m^2^) with those classified as ‘normal’ (BMI > 18.5 kg/m^2^ and < 25 kg/m^2^).

### Body mass index and initial weight status

Self-reported height and weight were used to calculate BMI for each participant. BMI was considered in its continuous form for the outcome variable. Initial weight status was the BMI category at baseline (in 2007).

### Weight related perceptions

Two questions on weight-related perceptions were asked in the 2007 survey. The first asked “How satisfied are you with your current weight”. Answers were ‘very satisfied’, ‘satisfied’, ‘neither satisfied nor dissatisfied’, ‘dissatisfied’, ‘very dissatisfied’ or ‘refused/not stated’. We classified these answers into ‘dissatisfied’ *(‘dissatisfied’, ‘very dissatisfied’)*, ‘not dissatisfied/ambivalent’ *(‘very satisfied’, ‘satisfied’, ‘neither satisfied nor dissatisfied’)* or ‘other’ *(‘refused/not stated’).* The second indicator was self-rated weight status, as follows: “Do you consider yourself to be… acceptable weight / underweight / overweight?” Answers were classified into ‘acceptable’, ‘overweight’ or ‘other’.

### Neighbourhood socioeconomic disadvantage

The Australian Bureau of Statistics (ABS) Socio Economic Indices For Areas (SEIFA) was used to measure neighbourhood socioeconomic disadvantage. The index selected for this study was of relative disadvantage, a composite indicator derived via principal components analysis by the Australian Bureau of Statistics to summarise multiple census data on income, education, employment, occupation, housing and other indicators of relative disadvantage (e.g. no car ownership) [44]. Lower values on this index denote an increasing concentration of disadvantaged people. As our focus was on disadvantaged communities and required stratification for purposes of comparing associations between weight change and weight-related (mis)perceptions across different levels of neighbourhood socioeconomic circumstances, this variable was inverted and then classified into tertiles so that higher strata denoted more disadvantaged areas. Our previous work [3, 45, 46] and that of others internationally [47] has shown that residents (especially women) in socioeconomically disadvantaged neighbourhoods tend to have higher BMI on average and independent of related factors such as educational attainment, employment status and household income. In this study, this measure of neighbourhood socioeconomic circumstances was utilised as a potential effect modifier, to reveal plausible differences in weight change trajectories over time between people living in disadvantaged and affluent areas with different stated perceptions of their weight at baseline.

### Potential confounding variables

A range of variables were identified to reduce probable sources of confounding based upon a synthesis of previous literature [47–49]. These included age, whether a participant was living on their own or as part of a couple (married or cohabiting), the highest level of education achieved (less than high school, high school to advanced diploma, university or higher), average household gross income (expressed in quintiles), the percentage of time in the last year spent unemployed, and geographic remoteness. Geographic remoteness was measured using the Accessibility/Remoteness Index of Australia (ARIA) [50], which helped to distinguish between participants living in urban areas (defined by the “major city” category) and or regional and remote areas (defined by those living in “inner regional”, “outer regional”, “remote” and “very remote” areas of Australia).

### Analytical strategy

The sample was described using cross-tabulations and mean BMIs at baseline and follow-up for each covariate. Multilevel linear regression models were used to investigate associations between BMI trajectories between baseline and follow-up with respect to neighbourhood disadvantage, initial weight status and the perceived weight status variables, adjusting for confounders. This was implemented by cross-classifying actual weight status at baseline with the weight-related perception variables, then fitting two-way interaction terms followed by a three-way interaction term between this cross-classified actual vs. perceived weight variable, neighbourhood disadvantage and time. These models were fitted separately for men and women due to known differences in risk of experiencing weight-related discrimination. The multilevel models were used to take account clustering of participants (level 1) within households (level 2) and areas of residence (level 3) as reported at baseline. Neighbourhoods were defined as Census Collection Districts (‘CCDs’), which are small areas containing approximately 225 residential dwellings on average. Results were presented using adjusted predicted mean BMI trajectories with 95% confidence intervals (95%CIs) from these models. All analyses were conducted in MLwIN v2.30 [51]. Ethical approval for the HILDA study was obtained from the Faculty of Business and Economics Human Ethics Advisory Committee at the University of Melbourne. Approval for the use of HILDA data was provided by the Government Department of Social Services.

## Results

Table 1 shows mean BMI was slightly higher in more disadvantaged neighbourhoods among men (correlation coefficient = 0.07, *p* < 0.0001) and especially women (0.12, *p* < 0.0001). BMI tended to be appreciably higher among participants reporting dissatisfaction with their weight (correlation coefficient: men = 0.37, *p* < 0.0001; women = 0.41, *p* < 0.0001) and perceiving themselves as overweight (correlation coefficient: men = 0.54, *p* < 0.0001; women = 0.55, *p* < 0.0001). Mean BMI varied by age, couple status, highest educational qualification and percentage of the previous year spent unemployed, but less so across quintiles of annual household income and the geographic remoteness of the place of residence.

**Table 1:** Description of the study sample at baseline (wave 7) and 5-year follow-up (wave 12)

	Men			Women		
	N (%)	Body Mass Index (mean)		N	Body Mass Index (mean)	
	(Baseline)	Baseline	5-year follow-up	(Baseline)	Baseline	5-year follow-up
N	4386	26.9	27.4	3788	26.1	26.8
Disadvantage tertiles						
Affluent	1381 (36.5%)	26.4	26.9	1561 (35.6%)	25.2	25.9
Average	1261 (33.3%)	27.0	27.8	1494 (34.1%)	26.2	26.9
Disadvantaged	1146 (30.3%)	27.3	27.7	1331 (30.4%)	27.1	27.6
Dissatisfied						
No/ambivalent	2640 (69.7%)	25.6	26.3	2422 (55.2%)	23.9	24.7
Yes	1138 (30.0%)	29.8	30.1	1940 (44.2%)	29.0	29.5
Missing	10 (0.3%)	26.9	27.5	24 (0.6%)	25.0	26.6
Perception						
Acceptable	2174 (57.4%)	25.0	25.7	2290 (52.2%)	23.2	24.0
Overweight	1391 (36.7%)	30.6	30.9	1942 (44.3%)	30.0	30.5
Missing	223 (5.9%)	21.2	22.6	154 (3.5%)	20.2	21.4
Age group						
15–24	557 (14.7%)	23.9	25.2	671 (15.3%)	23.5	24.4
25–34	549 (14.5%)	26.7	26.4	675 (15.4%)	25.8	25.9
35–44	732 (19.3%)	27.3	27.9	851 (19.4%)	26.4	27.1
45–54	797 (21.0%)	27.7	28.1	855 (19.5%)	26.8	27.1
55–64	590 (15.6%)	28.1	28.2	693 (15.8%)	27.7	27.9
65–74	404 (10.7%)	27.1	27.9	417 (9.5%)	26.5	27.5
75+	159 (4.2%)	26.2	26.1	224 (5.1%)	26.2	26.0
Couple status						
Yes	2652 (70.0%)	27.4	27.7	2814 (64.2%)	26.4	26.9
No	1136 (30.0%)	25.7	26.8	1571 (35.8%)	25.6	26.6
Refused	0 (0.0%)	0.0	0.0			
Education						
<=year 11%	1044 (27.6%)	26.8	28.0	1664 (37.9%)	26.5	27.7
Year 12 to adv diploma%	1892 (50.0%)	27.0	27.5	1676 (38.2%)	26.2	26.8
university	850 (22.4%)	26.6	26.8	1045 (23.8%)	25.4	25.8
undetermined	2 (0.1%)	26.3	26.8	1 (0.0%)	24.3	23.4
Percent unemployed						
0%	3558 (93.9%)	26.9	27.4	4090 (93.3%)	26.1	26.8
1–24%	95 (2.5%)	26.2	28.4	112 (2.6%)	25.3	25.9
25–49%	47 (1.2%)	25.3	26.7	68 (1.6%)	25.5	28.0
50–74%	36 (1.0%)	26.0	27.1	45 (1.0%)	28.0	25.7
75–100%	52 (1.4%)	26.0	26.8	71 (1.6%)	27.2	27.8
Income quintiles						
1 (low)	745 (19.7%)	27.1	27.2	1082 (24.7%)	26.8	27.1
2	840 (22.2%)	26.7	27.6	976 (22.3%)	26.3	27.1
3	902 (23.8%)	26.8	27.4	951 (21.7%)	26.4	27.1
4	729 (19.2%)	27.0	27.6	787 (17.9%)	25.6	26.9
5 (high)	572 (15.1%)	26.7	27.3	590 (13.5%)	24.9	26.0
Geographic remoteness						
Major city	2338 (61.7%)	26.7	27.3	2719 (62.0%)	26.0	26.5
Inner regional	961 (25.4%)	27.1	27.6	1112 (25.4%)	26.2	27.1
Outer regional	430 (11.4%)	27.2	28.0	482 (11.0%)	26.9	27.5
remote	59 (1.6%)	27.5	28.2	73 (1.7%)	26.3	27.6

Statistically significant (*p* < 0.001) chi-square values of the weight-related perception and satisfaction variables, both overall and for men and women separately, suggested an imperfect correlation between perceived overweight and dissatisfaction with current weight status. Table 2 shows the extent that unadjusted mean BMI for men and women at baseline varied with respect to a cross-classification of actual weight status and perception of weight. Among men with a ‘normal’ BMI by WHO criteria, those expressing dissatisfaction with their weight had a lower BMI compared to their peers who did not. The opposite pattern was observed for women of ‘normal’ BMI. The perception of being overweight was associated with higher BMI for men and women regardless of whether they were actually overweight or ‘normal’. Some of these patterns at baseline appeared to vary by neighbourhood disadvantage. For example, men expressing dissatisfaction despite having ‘normal’ weight had lower BMI if living in a disadvantaged neighbourhood (mean = 20.7 kg/m^2^), compared to their peers in the same BMI category also expressing dissatisfaction but living in more affluent areas (mean = 22.2 kg/m^2^).

**Table 2:** Distribution of weight status, dissatisfaction and perception of weight status and mean body mass index at baseline, by gender and neighbourhood disadvantage

	MEN								WOMEN							
			Neighbourhood Disadvantage (tertiles)								Neighbourhood Disadvantage (tertiles)					
	Total		Affluent		Average		Disadvantaged		Total		Affluent		Average		Disadvantaged	
	N	BMI (mean)	N	BMI (mean)	N	BMI (mean)	N	BMI (mean)	N	BMI (mean)	N	BMI (mean)	N	BMI (mean)	N	BMI (mean)
Weight status-Dissatisfied																
Normal-No/Ambivalent	1238	22.6	488	22.6	409	22.6	341	22.5	1657	21.6	656	21.5	546	21.6	455	21.6
Overweight-No/Ambivalent	1402	28.3	449	27.7	469	28.5	484	28.7	765	28.8	212	27.9	265	28.9	288	29.5
Normal-Dissatisfied	154	21.6	52	22.2	49	22.0	53	20.7	514	22.7	225	22.9	170	22.6	119	22.6
Overweight-Dissatisfied	984	31.1	390	30.2	329	31.2	265	32.1	1426	31.2	462	30.4	504	31.2	460	32.1
Other	10	26.9	2	27.2	5	26.7	3	27.0	24	25.0	6	24.3	9	23.4	9	27.0
Weight status-Perception																
Normal-Acceptable	1134	22.8	449	22.8	376	22.8	309	22.7	1707	21.8	708	21.7	563	21.7	436	21.9
Overweight-Acceptable	1040	27.5	338	27.1	356	27.7	346	27.7	583	27.6	167	27.0	204	27.5	212	28.1
Normal-Overweight	59	23.7	26	23.7	19	24.1	14	22.9	341	23.4	143	23.2	114	23.5	84	23.5
Overweight-Overweight	1332	30.9	498	30.0	436	31.2	398	31.8	1601	31.4	507	30.5	565	31.4	529	32.3
Other	223	21.2	70	20.9	74	21.7	79	21.0	154	20.2	36	19.4	48	19.6	70	21.0

*BMI* Body Mass Index

**Fig. 1:**
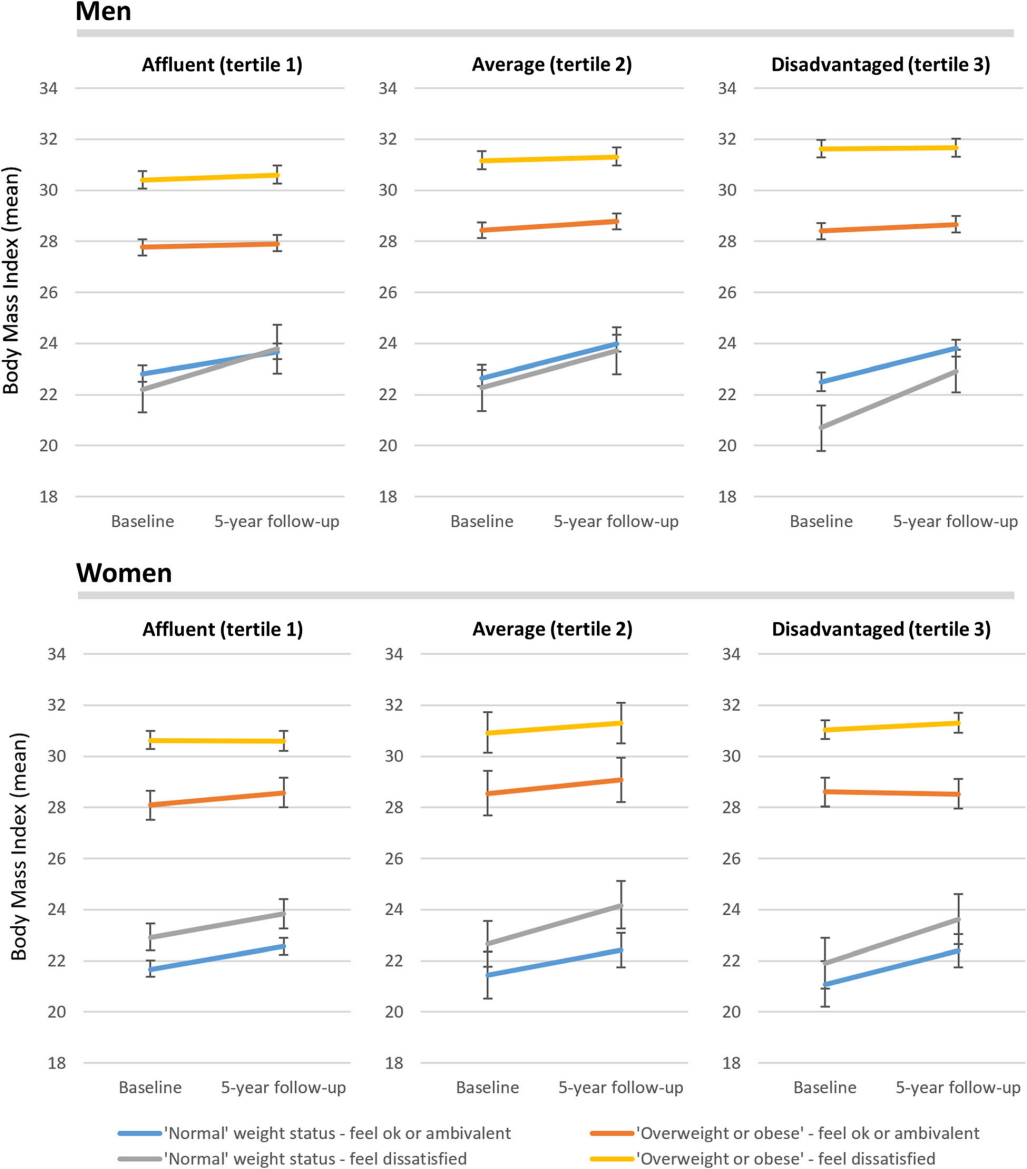
Predicted mean body mass index trajectories over 5-years with 95% confidence intervals for gender-stratified multilevel models with interaction terms between time, neighbourhood disadvantage, dissatisfaction with weight status and actual weight status (both measured at baseline), adjusted for age group, couple status, highest educational qualification, percentage of the last 12 months spent unemployed, annual household income, and geographic remoteness

**Fig. 2:**
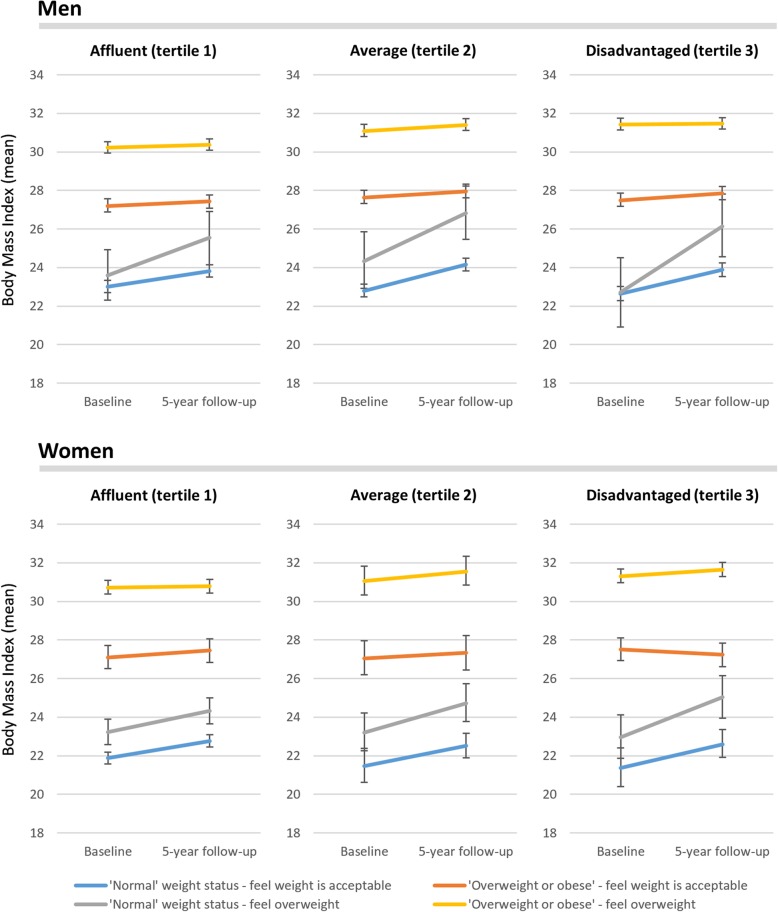
Predicted mean body mass index trajectories over 5-years with 95% confidence intervals for gender-stratified multilevel models with interaction terms between time, neighbourhood disadvantage, perception of weight status and actual weight status (both measured at baseline), adjusted for age group, couple status, highest educational qualification, percentage of the last 12 months spent unemployed, annual household income, and geographic remoteness

Predicted mean BMI trajectories and 95% confidence intervals from gender-stratified fully adjusted multilevel models with interactions between time, neighbourhood disadvantage and the cross-classification of baseline weight status and weight-related satisfaction and are shown in Fig. 1. Figure 2 shows virtually the same models, except perceived weight status was substituted in for weight-related satisfaction. Both figures reveal a complex range of trajectories in BMI change across these groups. The multilevel models on which the means were predicted for Figs. 1 and 2 are provided in Additional file 1: Table S1 and Additional file 2: Table S2, respectively.

In Fig. 1, men and women who were already overweight did not appear to gain or lose much weight between baseline and follow-up. This was regardless of whether participants felt dissatisfied with their weight or not. In contrast, Fig. 1 also shows gains in weight among men and women who began the study having ‘normal’ weight by WHO criteria. These gains were observed for persons who felt dissatisfied with their weight and those who did not. These patterns were consistent across strata of neighbourhood socioeconomic disadvantage.

In Fig. 2, as with Fig. 1, people who were overweight at baseline tended not to gain or lose weight on average across the 5 years. Weight gain was seen among people who had a ‘normal’ BMI by WHO criteria at baseline. Weight gain was observed for people who perceived themselves as being overweight, especially among men and women in disadvantaged neighbourhoods. People who felt their weight was acceptable gained weight.

## Discussion

In our study, the first notable finding is that among people who were overweight or obese at baseline, people did not gain or lose weight (on average) regardless of whether they perceived themselves as overweight or not. The same results were observed when focussing on whether a person expressed dissatisfaction with their weight at baseline. Second, weight gain was more common for men and women who had a ‘normal’ weight status at baseline by WHO criteria, but a little more rapid for those who perceived themselves to be overweight or dissatisfied with their weight. Third, this weight gain among people who over-perceived their ‘normal’ weight status was greater for those living in disadvantaged neighbourhoods.

Our findings reflect an interplay between actual weight status, perceived weight status and neighbourhood disadvantage. It is not fully clear at the present time what explains these findings, though it may be helpful to rule some explanations out. First, people who under-perceived their overweight did not substantially gain more weight, though neither did they appear to lose weight on average. Some previous studies have suggested the possibility of a protective mis-perception of weight status among children [18, 19], wherein under-perception of overweight may help reduce the probability of gaining weight over time (or be indicative of other, unmeasured, protective factors). As little change occurred among people who were overweight regardless of their perception or level of satisfaction, this does not provide evidence to support the protective mis-perception hypothesis. Nor does it provide evidence to support the mainstreaming of attempts to correct for under-perceptions of overweight status. Additionally, visual normalisation of overweight may not only make it acceptable within certain contexts to be heavier by raising the lower threshold of what is considered overweight [52–54], but it too may also provide an adjusted upper threshold as to what is generally felt to be too heavy for most people. This is an area that needs further research.

Our study does provide evidence for potentially correcting over-perceptions of weight status among people with a ‘normal’ BMI according to WHO criteria. Previous work has identified that people who perceive themselves as overweight have a higher probability of going on to gain weight [17], though fewer studies have differentiated between people who had a ‘normal’ BMI by WHO criteria from people who were actually overweight. A range of factors may be at play. First, regular exposure to media and advertising-driven portrayals of ideal body shapes and weights may be one reason why people may perceive themselves as overweight when they are not. Previous work has suggested that both men and (especially) women, tend to feel less satisfied with their bodies or be more likely to consider themselves overweight after viewing pictures of thin people (e.g. on television) [55–60].

These negative comparisons may spur a range of possible responses, some of which may be patterned by neighbourhood socioeconomic circumstances. One being emotional decision-making and engagement in maladaptive behaviours [26, 27] associated with ‘future discounting’. This is when people trade off the long-term consequences of alcohol and binge eating food packed with sugar and carbohydrates to satisfy short-term needs to mask feelings of shame, hopelessness and a lack of control [61, 62]. It is perhaps more likely to occur among people in disadvantaged areas who may feel they have few other options, while being encouraged by local obesogenic food environment providing numerous cues and opportunities to purchase junk food [41, 42]. By contrast, it is known that people in more affluent circumstances tend to have more healthier food environment (or at least healthier options) and be more likely to monitor their weight and engage in diet management [63].

Comfort eating of calorific food is compounded by the release of glucocorticoids that increases a person’s appetite for food that gives them pleasure under stressful situations [64, 65]. It is known that people in disadvantaged neighbourhoods experience chronic levels of stress more often than their peers in more affluent circumstances [66]. Although there were no consistent gender differences found in our study, women may be especially vulnerable to maladaptive behaviours [67–69], since there is greater value placed on female physical attractiveness [70] and a lower tolerance over minor weight gain among women than there is for men [71–73].

Further socioeconomic patterning may be driven by who or what people feel is the driver of their over-perceived weight status. An experimental study showed people with heavier BMI living in more affluent circumstances were more willing to excuse their body weight as something that was not entirely within their control, whereas people with higher BMI in more disadvantaged surroundings were more likely to blame themselves for their weight status [70]. Evidence also suggests that as body size increases, which will have been more dramatic among those in our study who had ‘normal’ BMI at baseline, women (but not men) tend to disconnect from social activities [74, 75], potentially increasing the risk of social isolation and associated behaviours such as eating disorders [76]. All of these factors may help to explain greater weight gain among people who over-perceived their weight status, and especially those in disadvantaged areas, though further research is warranted.

The study benefits from panel data over a 5-year period containing rich information, including a range of socioeconomic and demographic variables on a large number of men and women stratified by neighbourhood socioeconomic circumstances, WHO-defined BMI category and whether they perceived themselves as overweight and/or felt dissatisfied with their weight status. The longitudinal design afforded insights into different mean BMI trajectories over 5 years across all of these groups, potentially for the first time within a single study. The multilevel approach permitted a disentangling of effects between people and their places of residence and allowed assessment of change in BMI, while taking into account variation in mean baseline values between each group.

Some of the limitations and areas for future study include small sample sizes for ethnic groups, which would have been interesting to examine given prior work suggesting variations in weight-related perceptions between people of different ethnicities and countries of birth [77–80]. Another limitation is the known underreporting of weight and over-reporting of height [81], which means that the BMI variable used in our study can really only be considered a proxy for actual weight status. Studies that can replicate our longitudinal design but incorporate objectively measured BMI and/or other relevant outcome variables such as waist circumference and percentage abdominal fat would prove valuable next steps. It is important to note that this study is based upon observational data in which none of the perceptions of weight status, nor other variables analysed such as neighbourhood disadvantage, can be considered randomly assigned. As such, some residual confounding may remain even after multivariate adjustment. Finally, it would also be novel to examine not only whether mean BMI trajectories continue over a longer time-period among the groups in our study, but also to see at what point perceptions of weight change (if at all) over time.

## Conclusions

The findings of this study indicate, if interpreted in a causal sense and with caution, that practices to correct weight-related misperceptions may not be effective in promoting weight loss. We observed no weight loss (or gain) on average among people already overweight or obese at baseline regardless of their weight-related perception or satisfaction. In contrast, people who had a ‘normal’ weight at baseline by WHO criteria gained weight on average, especially among those who were dissatisfied with their weight, or who perceived themselves as overweight, in disadvantaged neighbourhoods. Randomised trials are warranted to examine the extent that these findings are robust to experimental design.

## Additional files

Additional file 1: **Table S1.** Multilevel models of five-year change in body mass index for men and women, accounting for potential effect modification of baseline actual weight status and satisfaction with weight status across strata of neighbourhood socioeconomic disadvantage. (DOCX 37 kb)

Additional file 2: **Table S2.** Multilevel models of five-year change in body mass index for men and women, accounting for potential effect modification of baseline actual weight status and perceived weight status across strata of neighbourhood socioeconomic disadvantage. (DOCX 37 kb)

## Abbreviations

ABS: Australian Bureau of Statistics
ARIA: Accessibility/Remoteness Index of Australia
BMI: Body mass index
CCD: Census Collection Districts
HILDA: Household, Income and Labour Dynamics in Australia
SEIFA: Socio Economic Indices For Areas
US: United States
WHO: World Health Organization
