# Supplementary material for: Does dissatisfaction with, or accurate perception of overweight status help people reduce weight? Longitudinal study of Australian adults
Source: BMC Public Health. 2019 May 22;19:619. doi: 10.1186/s12889-019-6938-3 (PMC6530191; doi:10.1186/s12889-019-6938-3)
Supplement: Supplementary file 1 — Table S1. Multilevel models of five-year change in body mass index for men and women, accounting for potential effect modification of baseline actual weight status and satisfaction with weight status across strata of neighbourhood socioeconomic disadvantage. (DOCX 37 kb) [file 12889_2019_6938_MOESM1_ESM.docx]

Table S1: Multilevel models of five-year change in body mass index for men and women, accounting for potential effect modification of baseline actual weight status and satisfaction with weight status across strata of neighbourhood socioeconomic disadvantage

|  |  |  |
| --- | --- | --- |
|  | **Men** | **Women** |
| Fixed Part | Coefficient (95%CI) p-value | Coefficient (95%CI) p-value |
| Constant | 22.74 (22.24, 23.23) p<0.001 | 21.59 (21.07, 22.12) p<0.001 |
|  |  |  |
| Time (ref: Baseline) |  |  |
| Follow-up | 0.86 (0.56, 1.16) p<0.001 | 0.89 (0.58, 1.21) p<0.001 |
|  |  |  |
| Neighbourhood Disadvantage (ref: Affluent) |  |  |
| Average | -0.18 (-0.59, 0.23) p=0.397 | -0.10 (-0.53, 0.34) p=0.665 |
| Disadvantaged | -0.32 (-0.77, 0.13) p=0.165 | 0.05 (-0.42, 0.51) p=0.840 |
|  |  |  |
| Weight Status and Weight Satisfaction (ref: 'Normal', Feel Satisfied/Ambivalent) |  |  |
| 'Normal', Feel Dissatisfied | -0.62 (-1.56, 0.33) p=0.201 | 1.25 (0.64, 1.87) p<0.001 |
| Overweight, Feel Satisfied/Ambivalent) | 4.96 (4.53, 5.40) p<0.001 | 6.43 (5.79, 7.06) p<0.001 |
| Overweight, Feel Dissatisfied | 7.59 (7.13, 8.05) p<0.001 | 8.97 (8.49, 9.46) p<0.001 |
|  |  |  |
| Time x Neighbourhood Disadvantage |  |  |
| Follow-up x Average | 0.50 (0.05, 0.95) p=0.031 | 0.09 (-0.38, 0.57) p=0.699 |
| Follow-up x Disadvantaged | 0.46 (-0.01, 0.92) p=0.054 | 0.41 (-0.08, 0.89) p=0.100 |
|  |  |  |
| Time x Weight Status and Satisfaction |  |  |
| Follow-up x 'Normal', Feel Dissatisfied | 0.72 (-0.27, 1.70) p=0.155 | 0.02 (-0.60, 0.65) p=0.939 |
| Follow-up x Overweight, Feel Satisfied/Ambivalent) | -0.72 (-1.15, -0.30) p=0.001 | -0.41 (-1.04, 0.21) p=0.194 |
| Follow-up x Overweight, Feel Dissatisfied | -0.66 (-1.10, -0.22) p=0.004 | -0.94 (-1.42, -0.46) p<0.001 |
|  |  |  |
| Weight Status and Satisfaction x Neighbourhood Disadvantage |  |  |
| 'Normal', Feel Dissatisfied x Average | 0.27 (-0.99, 1.53) p=0.674 | 0.00 (-0.87, 0.86) p=0.994 |
| 'Normal', Feel Dissatisfied x Disadvantaged | -1.17 (-2.46, 0.11) p=0.073 | -0.42 (-1.39, 0.54) p=0.391 |
| Overweight, Feel Satisfied/Ambivalent) x Average | 0.83 (0.25, 1.41) p=0.005 | 0.69 (-0.13, 1.52) p=0.101 |
| Overweight, Feel Satisfied/Ambivalent) x Disadvantaged | 0.95 (0.34, 1.56) p=0.002 | 1.10 (0.26, 1.93) p=0.010 |
| Overweight, Feel Dissatisfied x Average | 0.93 (0.31, 1.55) p=0.003 | 0.51 (-0.13, 1.16) p=0.120 |
| Overweight, Feel Dissatisfied x Disadvantaged | 1.53 (0.86, 2.21) p<0.001 | 0.98 (0.30, 1.66) p=0.005 |
|  |  |  |
| Time x Weight Status and Satisfaction x Neighbourhood Disadvantage |  |  |
| Time x 'Normal', Feel Dissatisfied x Average | -0.64 (-2.10, 0.83) p=0.394 | 0.48 (-0.47, 1.43) p=0.320 |
| Time x 'Normal', Feel Dissatisfied x Disadvantaged | 0.17 (-1.21, 1.56) p=0.808 | 0.40 (-0.62, 1.42) p=0.444 |
| Time x Overweight, Feel Satisfied/Ambivalent) x Average | -0.29 (-0.92, 0.34) p=0.363 | -0.04 (-0.91, 0.83) p=0.932 |
| Time x Overweight, Feel Satisfied/Ambivalent) x Disadvantaged | -0.35 (-0.98, 0.28) p=0.276 | -0.97 (-1.84, -0.11) p=0.027 |
| Time x Overweight, Feel Dissatisfied x Average | -0.55 (-1.22, 0.12) p=0.107 | 0.35 (-0.35, 1.05) p=0.330 |
| Time x Overweight, Feel Dissatisfied x Disadvantaged | -0.61 (-1.31, 0.08) p=0.084 | -0.10 (-0.81, 0.61) p=0.782 |
|  |  |  |
| Age Group (ref: 18-24) |  |  |
| 25-34 | 0.65 (0.34, 0.97) p<0.001 | 0.35 (0.01, 0.69) p=0.043 |
| 35-44 | 0.74 (0.40, 1.09) p<0.001 | 0.64 (0.27, 1.00) p=0.001 |
| 45-54 | 0.79 (0.44, 1.14) p<0.001 | 0.71 (0.34, 1.08) p<0.001 |
| 55-64 | 0.66 (0.29, 1.03) p<0.001 | 0.76 (0.36, 1.15) p<0.001 |
| 65-74 | 0.59 (0.19, 1.00) p=0.004 | 0.41 (-0.03, 0.85) p=0.070 |
| 75+ | -0.36 (-0.85, 0.12) p=0.138 | -0.11 (-0.63, 0.42) p=0.689 |
|  |  |  |
| Couple Status (ref: In A Couple) |  |  |
| Not In A Couple | -0.15 (-0.36, 0.06) p=0.160 | -0.38 (-0.60, -0.16) p=0.001 |
| Refused | 1.96 (-0.56, 4.47) p=0.128 | 0.68 (-2.35, 3.71) p=0.660 |
|  |  |  |
| Highest Educational Qualification (ref: School) |  |  |
| Year 12 to Advanced Diploma | -0.12 (-0.35, 0.11) p=0.300 | -0.04 (-0.29, 0.20) p=0.726 |
| University | -0.80 (-1.10, -0.50) p<0.001 | -0.30 (-0.61, 0.01) p=0.056 |
| Undetermined | -0.95 (-5.48, 3.57) p=0.680 | 1.42 (-6.20, 9.03) p=0.716 |
|  |  |  |
| Percentage Of Year Spent Unemployed (ref: 0%) |  |  |
| 1-24% | 0.19 (-0.25, 0.62) p=0.403 | 0.11 (-0.36, 0.59) p=0.636 |
| 25-49% | 0.00 (-0.58, 0.57) p=0.994 | 0.22 (-0.39, 0.83) p=0.480 |
| 50-74% | -0.24 (-0.93, 0.45) p=0.494 | 0.26 (-0.51, 1.03) p=0.512 |
| 75-100% | 0.18 (-0.39, 0.75) p=0.536 | 0.16 (-0.46, 0.78) p=0.607 |
|  |  |  |
| Annual Household Income (ref: Quintile 1) |  |  |
| Quintile 2 | -0.15 (-0.38, 0.09) p=0.218 | -0.30 (-0.56, -0.05) p=0.018 |
| Quintile 3 | -0.29 (-0.55, -0.04) p=0.024 | -0.25 (-0.53, 0.03) p=0.080 |
| Quintile 4 | -0.18 (-0.45, 0.09) p=0.188 | -0.20 (-0.50, 0.09) p=0.175 |
| Quintile 5 | -0.10 (-0.38, 0.19) p=0.512 | -0.36 (-0.67, -0.04) p=0.025 |
|  |  |  |
| Geographic Remoteness (ref: Major City) |  |  |
| Inner Regional | -0.10 (-0.32, 0.13) p=0.405 | -0.05 (-0.30, 0.21) p=0.721 |
| Outer Regional | 0.03 (-0.28, 0.35) p=0.829 | 0.42 (0.07, 0.78) p=0.020 |
| Remote, or Very Remote | -0.33 (-1.05, 0.40) p=0.375 | 0.22 (-0.58, 1.02) p=0.590 |
|  |  |  |
| Random Part | Variance (95%CI) | Variance (95%CI) |
| Level 4: Census Collection District (baseline) | 0.00 (0.00, 0.00) | 0.79 (0.20, 1.37) |
| Level 3: Household (baseline) | 1.29 (-0.13, 2.71) | 1.78 (-0.16, 3.73) |
| Level 2: Person | 6.77 (5.30, 8.24) | 8.83 (6.90, 10.76) |
| Level 1: Time | 5.05 (4.83, 5.28) | 7.38 (7.07, 7.69) |
|  |  |  |
| Number of units |  |  |
| Level 4: Census Collection District (baseline) | 2528 | 2830 |
| Level 3: Household (baseline) | 3592 | 4165 |
| Level 2: Person | 3788 | 4386 |
| Level 1: Time | 7576 | 8772 |
| 95%CI: 95% Confidence Interval |  |  |
