# Supplementary material for: Does dissatisfaction with, or accurate perception of overweight status help people reduce weight? Longitudinal study of Australian adults
Source: BMC Public Health. 2019 May 22;19:619. doi: 10.1186/s12889-019-6938-3 (PMC6530191; doi:10.1186/s12889-019-6938-3)
Supplement: Supplementary file 2 — Table S2. Multilevel models of five-year change in body mass index for men and women, accounting for potential effect modification of baseline actual weight status and perceived weight status across strata of neighbourhood socioeconomic disadvantage. (DOCX 37 kb) [file 12889_2019_6938_MOESM2_ESM.docx]

Table S2: Multilevel models of five-year change in body mass index for men and women, accounting for potential effect modification of baseline actual weight status and perceived weight status across strata of neighbourhood socioeconomic disadvantage

|  |  |  |
| --- | --- | --- |
|  | **Men** | **Women** |
| Fixed Part | Coefficient (95%CI) p-value | Coefficient (95%CI) p-value |
| Constant | 23.01 (22.52, 23.50) p<0.001 | 21.96 (21.45, 22.47) p<0.001 |
|  |  |  |
| Time (ref: Baseline) |  |  |
| Follow-up | 0.80 (0.49, 1.12) p<0.001 | 0.88 (0.57, 1.18) p<0.001 |
|  |  |  |
| Neighbourhood Disadvantage (ref: Affluent) |  |  |
| Average | -0.22 (-0.64, 0.21) p=0.321 | -0.16 (-0.58, 0.26) p=0.454 |
| Disadvantaged | -0.37 (-0.83, 0.09) p=0.114 | 0.03 (-0.43, 0.48) p=0.914 |
|  |  |  |
| Weight Status and Weight Perception (ref: 'Normal', Feel Acceptable) |  |  |
| 'Normal', Feel Overweight | 0.58 (-0.74, 1.90) p=0.390 | 1.36 (0.64, 2.07) p<0.001 |
| Overweight, Feel Acceptable | 4.19 (3.72, 4.65) p<0.001 | 5.22 (4.54, 5.89) p<0.001 |
| Overweight, Feel Overweight | 7.20 (6.78, 7.63) p<0.001 | 8.85 (8.40, 9.31) p<0.001 |
|  |  |  |
| Time x Neighbourhood Disadvantage |  |  |
| Follow-up x Average | 0.54 (0.07, 1.01) p=0.024 | 0.17 (-0.29, 0.63) p=0.472 |
| Follow-up x Disadvantaged | 0.43 (-0.06, 0.92) p=0.082 | 0.37 (-0.12, 0.85) p=0.139 |
|  |  |  |
| Time x Weight Status and Weight Perception |  |  |
| Follow-up x 'Normal', Feel Overweight | 1.15 (-0.18, 2.49) p=0.091 | 0.22 (-0.51, 0.95) p=0.551 |
| Follow-up x Overweight, Feel Acceptable | -0.58 (-1.05, -0.11) p=0.015 | -0.50 (-1.19, 0.18) p=0.148 |
| Follow-up x Overweight, Feel Overweight | -0.64 (-1.07, -0.22) p=0.003 | -0.82 (-1.28, -0.36) p<0.001 |
|  |  |  |
| Weight Status and Weight Perception x Neighbourhood Disadvantage |  |  |
| 'Normal', Feel Overweight x Average | 0.94 (-0.97, 2.85) p=0.336 | 0.37 (-0.65, 1.39) p=0.478 |
| 'Normal', Feel Overweight x Disadvantaged | -0.50 (-2.64, 1.64) p=0.649 | 0.25 (-0.87, 1.37) p=0.667 |
| Overweight, Feel Acceptable x Average | 0.66 (0.03, 1.29) p=0.039 | 0.37 (-0.52, 1.25) p=0.418 |
| Overweight, Feel Acceptable x Disadvantaged | 0.67 (0.01, 1.33) p=0.046 | 0.94 (0.03, 1.84) p=0.042 |
| Overweight, Feel Overweight x Average | 1.09 (0.50, 1.67) p<0.001 | 0.74 (0.13, 1.36) p=0.018 |
| Overweight, Feel Overweight x Disadvantaged | 1.58 (0.96, 2.20) p<0.001 | 1.09 (0.44, 1.74) p=0.001 |
|  |  |  |
| Time x Weight Status and Weight Perception x Neighbourhood Disadvantage |  |  |
| Time x 'Normal', Feel Overweight x Average | -0.01 (-2.06, 2.03) p=0.990 | 0.27 (-0.84, 1.37) p=0.637 |
| Time x 'Normal', Feel Overweight x Disadvantaged | 1.02 (-1.20, 3.23) p=0.369 | 0.60 (-0.59, 1.79) p=0.324 |
| Time x Overweight, Feel Acceptable x Average | -0.46 (-1.14, 0.23) p=0.195 | -0.25 (-1.20, 0.70) p=0.610 |
| Time x Overweight, Feel Acceptable x Disadvantaged | -0.29 (-0.98, 0.41) p=0.420 | -1.00 (-1.95, -0.06) p=0.038 |
| Time x Overweight, Feel Overweight x Average | -0.40 (-1.03, 0.24) p=0.224 | 0.28 (-0.39, 0.96) p=0.405 |
| Time x Overweight, Feel Overweight x Disadvantaged | -0.55 (-1.20, 0.11) p=0.101 | -0.08 (-0.77, 0.61) p=0.813 |
|  |  |  |
| Age Group (ref: 18-24) |  |  |
| 25-34 | 0.70 (0.39, 1.01) p<0.001 | 0.34 (0.01, 0.68) p=0.044 |
| 35-44 | 0.73 (0.39, 1.07) p<0.001 | 0.58 (0.22, 0.94) p=0.002 |
| 45-54 | 0.69 (0.34, 1.03) p<0.001 | 0.61 (0.25, 0.98) p=0.001 |
| 55-64 | 0.51 (0.14, 0.87) p=0.006 | 0.57 (0.18, 0.95) p=0.004 |
| 65-74 | 0.41 (0.02, 0.81) p=0.040 | 0.25 (-0.18, 0.68) p=0.262 |
| 75+ | -0.53 (-1.00, -0.06) p=0.028 | -0.14 (-0.65, 0.37) p=0.590 |
|  |  |  |
| Couple Status (ref: In A Couple) |  |  |
| Not In A Couple | -0.11 (-0.32, 0.10) p=0.305 | -0.42 (-0.64, -0.20) p<0.001 |
| Refused | 1.68 (-0.82, 4.18) p=0.189 | 0.73 (-2.28, 3.74) p=0.635 |
|  |  |  |
| Highest Educational Qualification (ref: School) |  |  |
| Year 12 to Advanced Diploma | -0.14 (-0.37, 0.09) p=0.231 | -0.02 (-0.27, 0.22) p=0.864 |
| University | -0.85 (-1.14, -0.55) p<0.001 | -0.33 (-0.63, -0.03) p=0.032 |
| Undetermined | -0.86 (-5.24, 3.52) p=0.700 | 1.31 (-6.08, 8.69) p=0.728 |
|  |  |  |
| Percentage Of Year Spent Unemployed (ref: 0%) |  |  |
| 1-24% | 0.19 (-0.25, 0.62) p=0.398 | 0.13 (-0.34, 0.60) p=0.596 |
| 25-49% | 0.06 (-0.51, 0.63) p=0.840 | 0.25 (-0.35, 0.86) p=0.409 |
| 50-74% | -0.28 (-0.96, 0.40) p=0.422 | 0.37 (-0.39, 1.14) p=0.340 |
| 75-100% | 0.26 (-0.31, 0.83) p=0.376 | 0.18 (-0.44, 0.79) p=0.570 |
|  |  |  |
| Annual Household Income (ref: Quintile 1) |  |  |
| Quintile 2 | -0.16 (-0.40, 0.07) p=0.162 | -0.35 (-0.60, -0.10) p=0.006 |
| Quintile 3 | -0.30 (-0.55, -0.04) p=0.021 | -0.31 (-0.58, -0.03) p=0.030 |
| Quintile 4 | -0.16 (-0.43, 0.10) p=0.220 | -0.27 (-0.56, 0.02) p=0.069 |
| Quintile 5 | -0.08 (-0.36, 0.20) p=0.582 | -0.43 (-0.75, -0.12) p=0.006 |
|  |  |  |
| Geographic Remoteness (ref: Major City) |  |  |
| Inner Regional | -0.11 (-0.33, 0.12) p=0.349 | -0.07 (-0.32, 0.18) p=0.600 |
| Outer Regional | 0.09 (-0.21, 0.40) p=0.552 | 0.32 (-0.03, 0.66) p=0.072 |
| Remote, or Very Remote | -0.28 (-0.99, 0.43) p=0.443 | 0.20 (-0.58, 0.98) p=0.615 |
|  |  |  |
| Random Part | Variance (95%CI) | Variance (95%CI) |
| Level 4: Census Collection District (baseline) | 0.03 (-0.34, 0.41) | 0.53 (0.01, 1.06) |
| Level 3: Household (baseline) | 0.66 (-0.73, 2.05) | 1.84 (0.00, 3.67) |
| Level 2: Person | 6.71 (5.31, 8.11) | 8.11 (6.29, 9.93) |
| Level 1: Time | 5.05 (4.82, 5.28) | 7.38 (7.07, 7.69) |
|  |  |  |
| Number of units |  |  |
| Level 4: Census Collection District (baseline) | 2528 | 2830 |
| Level 3: Household (baseline) | 3592 | 4165 |
| Level 2: Person | 3788 | 4386 |
| Level 1: Time | 7576 | 8772 |
| 95%CI: 95% Confidence Interval |  |  |
